# Delayed HLA-B27–Associated Uveitis Following Uncomplicated Phacovitrectomy: A Case Report

**DOI:** 10.7759/cureus.106173

**Published:** 2026-03-31

**Authors:** Kinga Jamontt, Malgorzata A Ozimek, Martyna Nowak, Barbara Roguska, Katarzyna Szlachetka, Julia Cholda, Magdalena Matlakiewicz, Wiktoria Janik, Agnieszka Kudasiewicz-Kardaszewska

**Affiliations:** 1 Ophthalmology, Professor Zagórski Eye Surgery Centre, OCHO Medical Group, Nowy Sącz, POL; 2 Ophthalmology, Medical University of Silesia, Katowice, POL; 3 Ophthalmology, The University Clinical Center of Professor K. Gibiński, Katowice, POL

**Keywords:** ankylosing spondylitis, hla-b27, intraocular surgery, phacovitrectomy, postoperative inflammation, uveitis

## Abstract

Postoperative inflammation after phacovitrectomy is typically mild, occurs early, and resolves with corticosteroid therapy. Delayed or recurrent uveitis following an initially uneventful postoperative course is uncommon and should prompt evaluation for non-infectious inflammatory causes, including underlying systemic disease. A 71-year-old man developed severe intraocular inflammation shortly after discontinuation of postoperative corticosteroids following uncomplicated phacovitrectomy. The early postoperative course was unremarkable. Repeated and detailed history taking revealed previously undisclosed ankylosing spondylitis with HLA-B27 positivity and a remote episode of iritis. The inflammatory flare was attributed to immune reactivation triggered by surgical stress and steroid withdrawal, necessitating intensive topical and systemic corticosteroid therapy. This case highlights the importance of thorough and repeated medical history assessment in patients with atypical postoperative inflammation. HLA-B27-associated disease should be considered in cases of delayed uveitis following intraocular surgery, as early recognition may influence perioperative management and reduce the risk of recurrent, vision-threatening complications.

## Introduction

Uveitis is a major cause of intraocular inflammation and a significant contributor to visual impairment worldwide [1,2]. It represents a heterogeneous group of disorders that can be classified anatomically into anterior, intermediate, posterior uveitis, and panuveitis, as well as etiologically into infectious and non-infectious forms [3]. Most cases are non-infectious and result from immune-mediated mechanisms influenced by genetic susceptibility and environmental triggers [4]. Among genetic factors associated with uveitis, human leukocyte antigen (HLA) alleles play a central role, particularly HLA-B27, which is strongly associated with acute anterior uveitis and systemic inflammatory diseases such as ankylosing spondylitis. HLA-B27-associated uveitis often follows a recurrent course and may remain clinically silent between flares, leading to delayed recognition of the underlying systemic condition. External factors, including surgical stress or withdrawal of immunosuppressive therapy, may precipitate inflammatory reactivation [5,6]. Combined phaco-vitrectomy is a widely performed and generally safe procedure in patients with concurrent anterior and posterior segment pathology [7]. In uncomplicated cases, postoperative inflammation is usually mild, occurs early, and resolves with topical corticosteroid therapy. Delayed or recurrent inflammation after an initially uneventful postoperative course is uncommon and should prompt further diagnostic evaluation beyond routine surgical causes [8].

This report describes a case of delayed postoperative uveitis following uncomplicated phaco-vitrectomy in which previously undisclosed HLA-B27-associated ankylosing spondylitis played a key role in the atypical inflammatory course. The case underscores the importance of repeated and thorough medical history taking in patients with unexpected postoperative inflammation and highlights implications for perioperative management and surgical planning of the fellow eye.

## Case presentation

A 71-year-old Caucasian male presented to the ophthalmology clinic with a gradual deterioration of vision in the right eye, accompanied by the perception of a “black floating object” and progressive visual distortion. His main subjective complaint was blurred vision described as foggy vision, along with worsening metamorphopsias, occurring exclusively in the right eye. The patient’s ocular history was notable for a contusion to the right eye approximately 1.5 years earlier, caused by a projectile from a toy pellet gun. This injury resulted in an intravitreal haemorrhage that was managed conservatively. The patient also had a history of mild open-angle glaucoma treated with topical 0.5% timolol eye drops.

**Figure 1:**
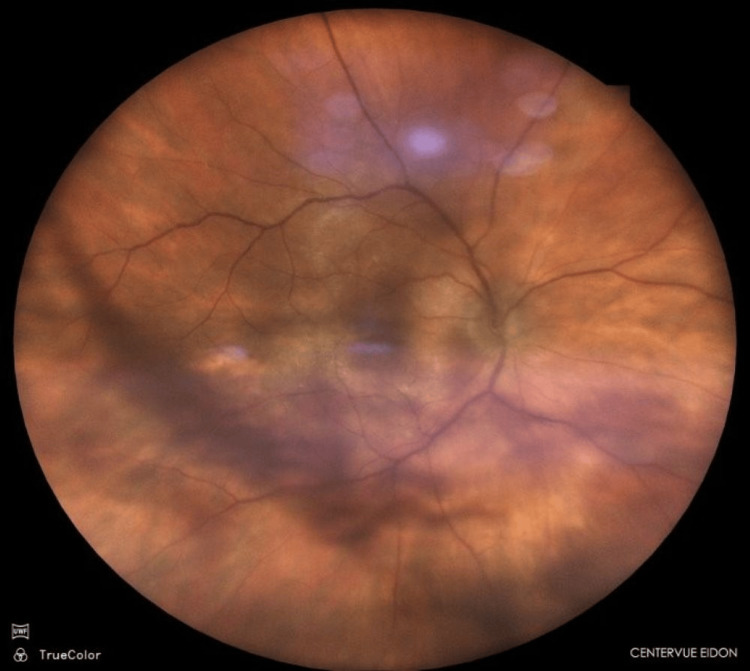
Fundus photograph of the right eye. Note – large floater obscuring the fundus appearance. (Fundus Camera – Centervue; ICare, Vantaa, Finland)

On initial examination, best-corrected, distant visual acuity (BCVA) in the right eye (RE) was 0.52 logMAR with correction (+2.5D/−0.75 D cyl × 90), while BCVA in the left eye (LE) was 0.8 with correction (+2.0 D). Intraocular pressure was 22 mmHg in the RE and 25 mmHg in the LE. Slit-lamp examination revealed clear corneas, normal iris configuration, and deep anterior chambers in both eyes. Meibomian gland dysfunction grade II/III was noted bilaterally. An incipient cataract was present in both eyes. Fundus examination of the right eye showed dense vitreous opacities corresponding to a large symptomatic floater located posterior to the crystalline lens, an optic disc cup-to-disc ratio of approximately 0.5, and an epiretinal membrane (ERM) involving the macula with associated retinal distortion (Figure 1).

**Figure 2:**
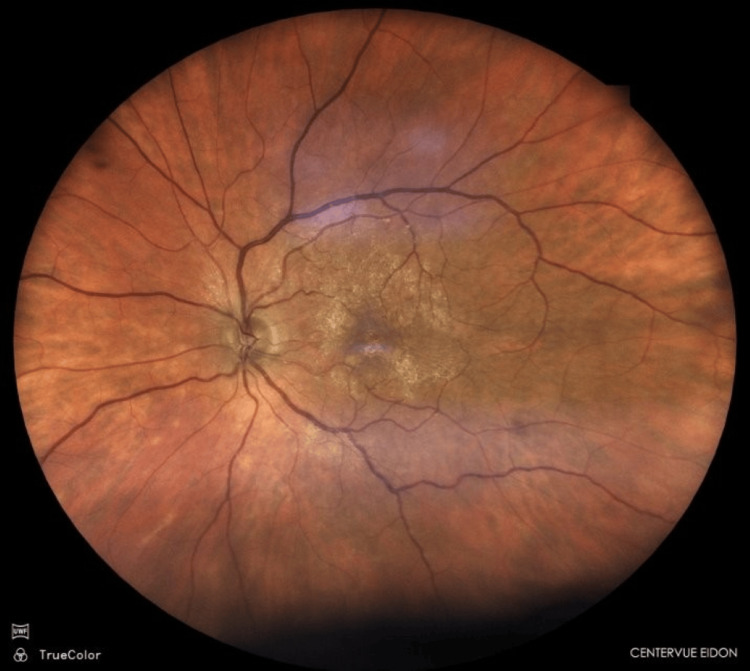
Fundus photograph of the left eye Fundus photograph of the left eye with asymptomatic epiretinal membrane in the macula.

In the left eye, posterior vitreous detachment was observed, the optic disc had a cup-to-disc ratio of approximately 0.5, and features consistent with a macular pucker were seen (Figure 2).

**Figure 3:**
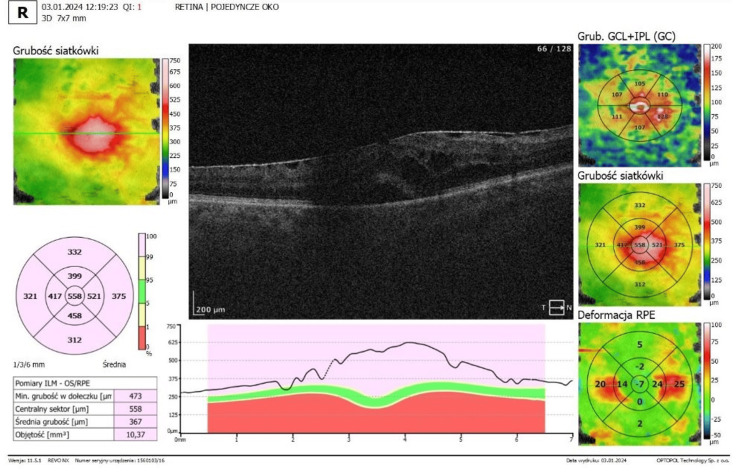
Optical coherence tomography (OCT) of the right eye OCT of the right eye, with epiretinal membrane and macular oedema. (SOCT; Optopol, Zawiercie, Poland)

**Figure 4:**
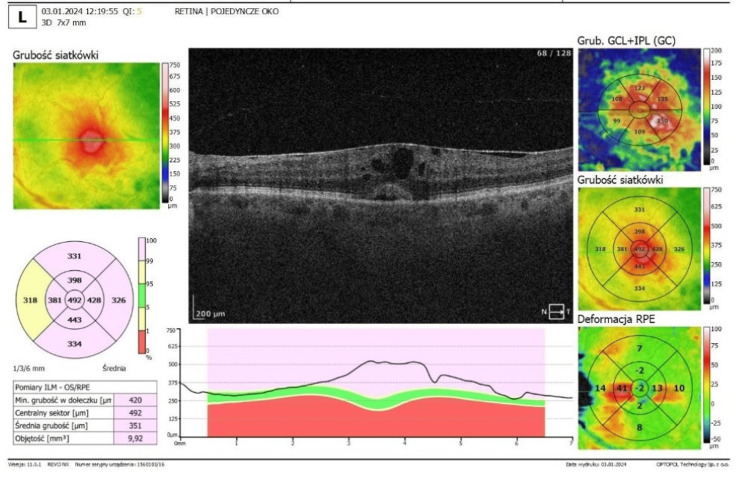
Optical coherence tomography (OCT) of the left eye OCT of the left eye with asymptomatic epiretinal membrane. Note – foveal flattening and macular oedema. (SOCT; Optopol, Zawiercie, Poland)

Optical coherence tomography (OCT) confirmed the presence of ERMs in both eyes. In the right eye, OCT demonstrated foveal flattening and distortion with associated retinal thickening and oedema (Figure 3). In the left eye, foveal flattening with mild retinal oedema secondary to the epiretinal membrane was also observed (Figure 4).

Given the coexistence of visually significant cataract and posterior segment pathology, combined phacoemulsification with intraocular lens (IOL) implantation and pars plana vitrectomy (phaco-vitrectomy) was recommended and performed first in the right eye.

**Figure 5:**
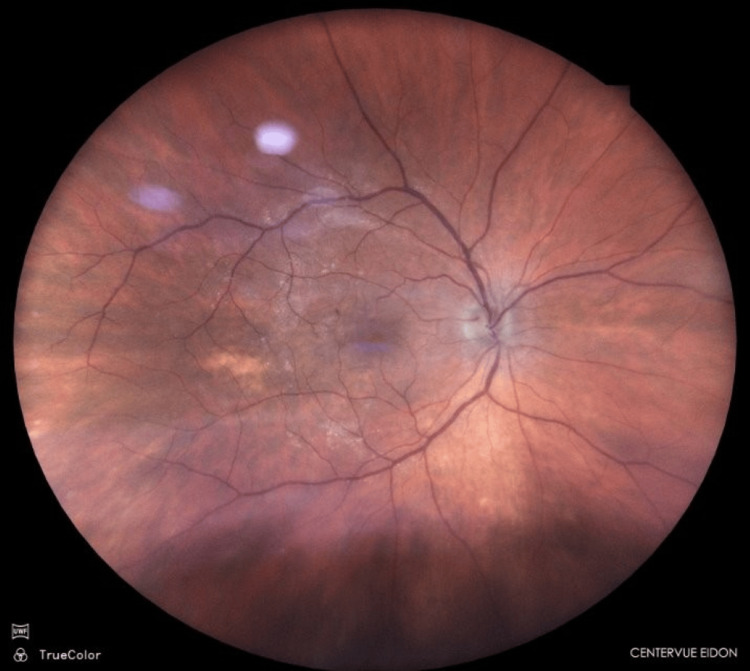
Fundus photograph Postoperative fundus photograph

**Figure 6:**
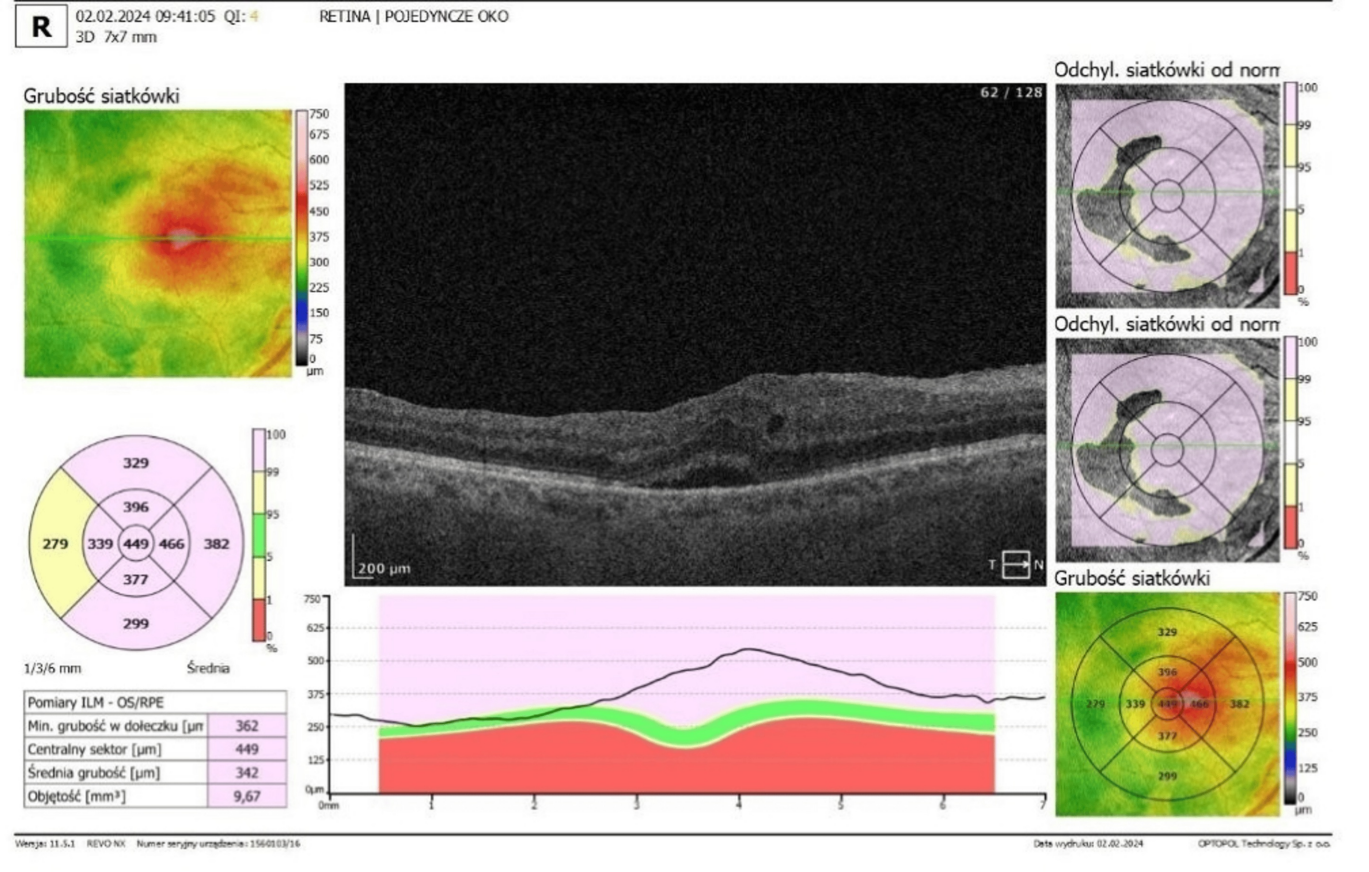
Optical coherence tomography (OCT) of the macula Postoperative OCT of the macula

The surgical procedure was uneventful, and the early postoperative course was unremarkable. Standard postoperative treatment was initiated: antibiotic/steroid drops four times a day for seven days (Ducressa; Santen, Helsinki, Finland), following topical steroid (0.1% dexamethasone; WZF, Warsaw, Poland) alone with slow tapering for the next four weeks. At the routine six-week postoperative follow-up visit, visual acuity improved, and the eye was quiet (Figures 5, 6). No signs of active inflammation were observed. Consequently, topical corticosteroid therapy was discontinued.

**Figure 7:**
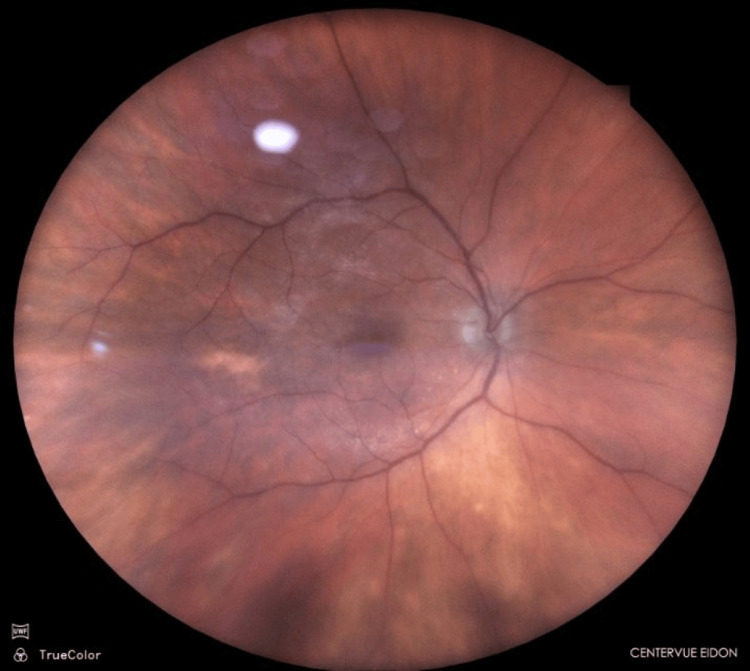
Fundus photograph Blurred fundus photograph due to recurrent inflammation.

**Figure 8:**
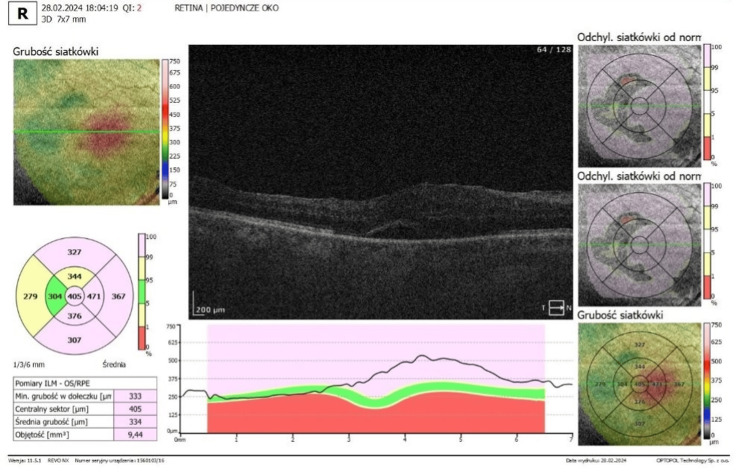
Optical coherence tomography (OCT) of the macula OCT of the macula two weeks after steroid cessation

Two weeks after steroid cessation, the patient returned with a sudden and significant deterioration of vision in the right eye. BCVA decreased to 1.3 logMAR. Slit-lamp and fundus examination revealed recurrent intraocular inflammation with fibrin exudate around the IOL and inflammatory vitreous opacities posterior to the IOL, with reduced fundus visibility (Figures 7, 8).

During this visit, further and more detailed past history revealed that the patient had been diagnosed several years earlier with ankylosing spondylitis. Additional testing confirmed HLA-B27 positivity. The patient also recalled a previous episode of iritis and choroiditis several years earlier, which had not been thoroughly investigated or treated.

**Figure 9:**
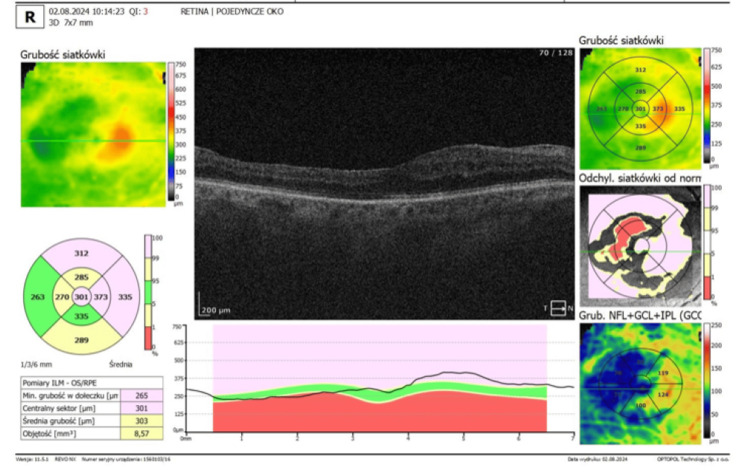
Optical coherence tomography (OCT) after second surgery - note complete resolution of subretinal fluid

**Figure 10:**
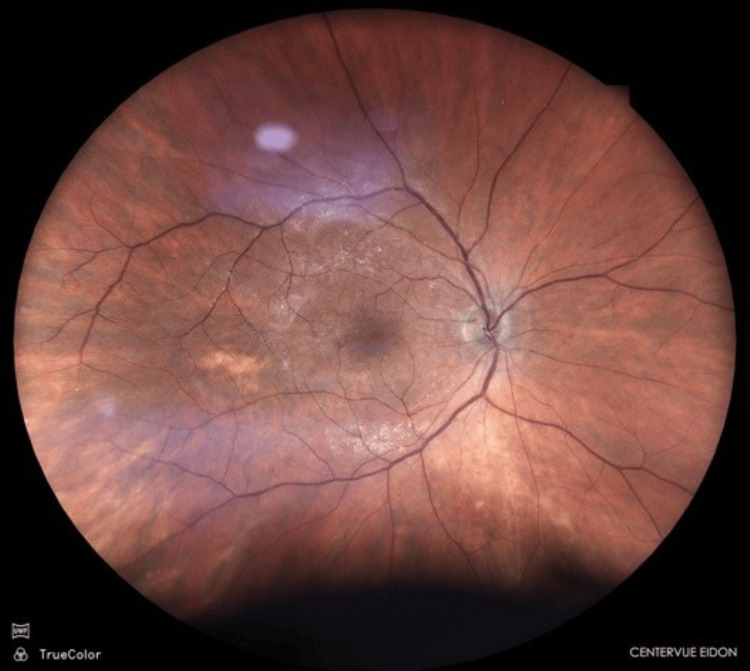
Fundus photograph after second surgery

Based on these findings, delayed postoperative uveitis was diagnosed in the context of previously diagnosed HLA-B27-positive ankylosing spondylitis, which had not been disclosed by the patient during the initial preoperative assessment. Intensive anti-inflammatory therapy was re-initiated, including topical and oral corticosteroids. Repeat pars plana vitrectomy was also performed [7]. Postoperative fundus OCT and fundus photo are depicted in Figures 9, 10.

At this stage, uncertainty remained regarding the optimal management of the fellow eye, which had not yet undergone surgery. For clarity, a summary of each stage in the patient's history is presented in Table 1.

**Table 1:** Summary of each stage in patient's medical history SRF - Subretinal fluid RE - right eye LE - left eye AC - anterior chamber PC - posterior chamber MGD - Meibomian gland dysfunction ERM - epiretinal membrane IOL - intraocular lens IOP - intraocular pressure PVD - posterior vitreous detachment BCVA - best-corrected visual acuity TPPV - through pars plana vitrectomy NSAID - nonsteroidal anti-inflammatory drugs

	First visit and treatment schedule	Post Phaco TPPV RE - Six weeks postop.	Two months postop (two weeks after steroid cessation)	NEXT STEP – reTPPV RE and postop.
Medical Interview	RE- vision deterioration, aggravating metamorphopsias, patient WANTS ”the best IOL in the world”	Visual acuity improvement postop.	Painless visual deterioration, fogging	Back to history – patient recalled that he had had the episode of iritis 3-4 years before, but no tests
BCVA	RE=0.3 cc +2.5/-0.75 cyl x 90; LE=0.8 cc +2.0	RE=0.6 sc; LE=0.6 cc +2.0	RE=0.05; LE=0.6	RE=0.8; LE=0.6 cc +2.5
Ocular Examination	RLE: MGD II/III, cornea clear, iris normal, deep AC, incipient cataract; RE: big floater behind the lens, disc 0.5, ERM on the macular distorting it; LE: PVD, disc 0.5, macular pucker	RE: cornea clear, AC clear and deep, PC-IOL in place, RE: Fundus – normal, retina attached, macula almost normal	RE: Little pink, cornea clear, tyndal(+), iris blurred, IOL coated with fibrin, visibility of the fundus reduced	RE: Calm, cornea clera, AC- clear, IOL clear no fibrin, no synechiae, fundus photo – normal, vitreous cavity clear
OCT	ERM+flatten and distorted fovea with oedema	no ERM, little SRF centrally, fovea flat	Macular oedema and SRF	Resolution of macular oedema
IOP	RE=22 mmHg; LE=25mmHg	RE=16 mmHg; LE=16 mmHg	RE=50 mmHg (!!!); LE=28 mmHg ON MEDICATIONS	RE=16 mmHg; LE=16 mmHg ON MEDICATIONS (timolol+dorzolamide+brimonidine)
Treatment	RE- phacoTPPV with monofocal lens LE- might need either phaco or phaco and TPPV Monitoring of glaucoma – perimetry, OCT, probably laser treatment postoperatively	Left on 0.5% timolol two times a day Scheduled for LE operation Discontinuation of steroids and NSAIDs	All medications EXCEPT FOR TIMOLOL were discontinued TWO WEEKS BEFORE pressure-lowering overload (acetazolamide, mannitol, timolol+dorzolamide) AND – UVEITIS LAB TESTS RE-TPPV SCHEDULED NEXT WEEK.	RE - TPPV done under general steroids Postop.- STEROIDS & ANTIBIOTICS Slow tapering – possible recurrence of uveitis

## Discussion

Combined phaco-vitrectomy is a well-established and widely performed procedure for concurrent anterior and posterior segment pathology [7]. In uncomplicated cases, postoperative inflammation is typically mild, occurs early, and resolves gradually with standard topical corticosteroid therapy [1]. Delayed or recurrent intraocular inflammation after an initially uneventful postoperative course is uncommon and generally prompts further investigation.

The present case illustrates an atypical postoperative course characterized by a delayed recurrence of intraocular inflammation following an initially favourable healing period. Importantly, the inflammatory flare occurred after tapering and discontinuation of corticosteroid therapy, which is not typical for routine postoperative inflammation after phaco-vitrectomy. Such a pattern should raise clinical suspicion and prompt a broader differential diagnosis, including infectious endophthalmitis and immune-mediated inflammation. In the absence of clinical or microbiological evidence of infection, a non-infectious inflammatory aetiology should be strongly considered.

HLA-B27-associated uveitis is a well-recognized extra-articular manifestation of ankylosing spondylitis and typically presents as an acute, recurrent anterior uveitis [5,6]. However, posterior segment involvement and more severe intraocular inflammation may occur, particularly in the context of systemic immune activation or surgical stress [9].

In this patient, the combination of ocular surgery and subsequent withdrawal of corticosteroid therapy likely acted as a trigger for immune reactivation, leading to delayed postoperative uveitis. Although the diagnosis of ankylosing spondylitis had been established previously, it was not disclosed during the initial ophthalmic evaluation, delaying recognition of the underlying systemic inflammatory background.

This case demonstrates the critical importance of repeated and detailed medical history assessment, particularly in patients presenting with atypical postoperative inflammation. Systemic inflammatory diseases may remain clinically silent for long periods or be underestimated by patients yet significantly influence postoperative outcomes. Recognition of the underlying systemic condition altered both diagnostic reasoning and therapeutic strategy, leading to prompt re-introduction of topical and systemic corticosteroids and consideration of further surgical intervention. 

Finally, this case raises important questions regarding the optimal management of the fellow eye. In patients with known HLA-B27-associated disease, careful preoperative risk assessment, interdisciplinary collaboration, and consideration of perioperative immunomodulatory strategies may be warranted to minimize the risk of postoperative inflammatory complications.

## Conclusions

Delayed postoperative uveitis after an initially uncomplicated phaco-vitrectomy is rare and should prompt evaluation for underlying systemic inflammatory disease. This case illustrates how previously undisclosed HLA-B27-associated ankylosing spondylitis can lead to atypical postoperative inflammation triggered by surgical stress and corticosteroid withdrawal. Thorough and repeated medical history assessment is essential for accurate diagnosis and timely management. Awareness of this association is crucial when planning surgical and therapeutic strategies, including surgery of the fellow eye, to reduce the risk of recurrent inflammation and vision-threatening complications.
